# Simple design of efficient broadband multifunctional polarization converter for X-band applications

**DOI:** 10.1038/s41598-021-81586-w

**Published:** 2021-01-21

**Authors:** Thi Kim Thu Nguyen, Thi Minh Nguyen, Hong Quang Nguyen, Thanh Nghia Cao, Dac Tuyen Le, Xuan Khuyen Bui, Son Tung Bui, Chi Lam Truong, Dinh Lam Vu, Thi Quynh Hoa Nguyen

**Affiliations:** 1 Graduate University of Science and Technology, Vietnam Academy of Science and Technology, 18 Hoang Quoc Viet, Cau Giay, Hanoi 100000 Vietnam; 2 School of Engineering and Technology, Vinh University, 182 Le Duan, Vinh City, 460000 Vietnam; 3 Department of Physics, Hanoi University of Mining and Geology, 18 Pho Vien, Hanoi, 100000 Vietnam; 4 Institute of Materials Science, Vietnam Academy of Science and Technology, 18 Hoang Quoc Viet, Cau Giay, Hanoi 100000 Vietnam; 5 NTT Hi-Tech Institute, Nguyen Tat Thanh University, Ho Chi Minh City, Vietnam

**Keywords:** Engineering, Materials science, Physics

## Abstract

A simple design of a broadband multifunctional polarization converter using an anisotropic metasurface for X-band application is proposed. The proposed polarization converter consists of a periodic array of the two-corner-cut square patch resonators based on the FR-4 substrate that achieves both cross-polarization and linear-to-circular polarization conversions. The simulated results show that the polarization converter displays the linear cross-polarization conversion in the frequency range from 8 to 12 GHz with the polarization conversion efficiency above 90%. The efficiency is kept higher than 80% with wide incident angle up to 45°. Moreover, the proposed design achieves the linear-to-circular polarization conversion at two frequency bands of 7.42–7.6 GHz and 13–13.56 GHz. A prototype of the proposed polarization converter is fabricated and measured, showing a good agreement between the measured and simulated results. The proposed polarization converter exhibits excellent performances such as simple structure, multifunctional property, and large cost-efficient bandwidth and wide incident angle insensitivity in the linear cross polarization conversion, which can be useful for X-band applications. Furthermore, this structure can be extended to design broadband polarization converters in other frequency bands.

## Introduction

The polarization is an essential parameter of the electromagnetic (EM) wave, mentioning the oscillating direction of the electric field in a plane orthogonal to the wave propagation direction^1^. Controlling the polarization state of the EM wave has attracted more and more research attention due to many polarization-sensitive applications and devices^2–8^. Among devices that exhibit polarization conversion control, polarization converter has been studied extensively for various applications such as improvement of antenna gain^10,11^, interference and radar cross section (RCS) reduction^12,13^, and stereoisomer identification^14^. Conventional polarization converters using the optical activity of crystals and the Faraday effect are narrow bandwidth, bulky volume, and incident angle dependence; thus, they are incompatible for real-world applications^15^. Therefore, the design of the polarization converter with simple structure, compact size, and good performances with a broad bandwidth and wide angular stability has remained challenging. Recently, many polarization converters based on the artificial two-dimensional planar metamaterial structure called metasurfaces (MTS) have been developed, providing a potential approach to manipulate the polarization state of EM waves through adjusting material parameters or changing the dimension and geometry of metasurface^16,17^. The MTS have demonstrated the ability of polarization control in different frequency ranges of the EM spectrum, such as the microwave^16–20^, terahertz^3,21^, infrared^22,23^, and visible^24,25^. However, the reported designs included single-layer^3,17,18,21–32^ and multi-layer^19,20,33–36^ only achieve high polarization conversion performance for either cross polarization converter (CPC)^3,18–35^ or linear-to-circular converter (LP-to-CP)^36–38^. More recently, many efforts have been made to develop a multifunctional polarization converter that exhibits both CPC and LP-to-CP conversion^39–44^ to miniature the size and reduce complexity and cost of the system^43^. Zheng et al. proposed a polarization converter that achieved CPC conversion in a lower band and LP-to-CP conversion in a higher band; however, it only operated efficiently for the normal incidence^39^. Furthermore, Ma et al. designed the multiband polarization converter composed of a two-corner-cut square disk surrounded by three concentric isomorphous rings that realized both CPC and LP-to-CP conversion in five frequency bands^40^. However, this design structure is only demonstrated for normal incidence and its operating frequency bands are almost not wide and/or polarization conversion efficiency is still low. Recently, Khan et al. reported a polarization converter that exhibited both CPC and LP-to-CP conversion; however, its incident angle stability is limited only for CPC conversion^42^. Therefore, the design of a single-layer multifunctional polarization converter with broad bandwidth and wide incidence angle stability for both CPC and LP-to-CP conversion is necessary to explore the potential practical applications.

In this paper, we propose a simple design of a broadband linear and circular polarization converter using an anisotropic metasurface for X-band applications. The proposed converter consists of a periodic array of a metallic two-corner-cut square patch resonator and a metallic ground plane separated by a dielectric substrate of FR-4. The polarization conversion performance and operation principle are thoroughly investigated. The simulation and experimental results confirm that the proposed design achieves linear polarization with the polarization conversion ratio (PCR) greater than 90% in the frequency range from 8 to 12 GHz. Moreover, this design can be obtained the linear-to-circular polarization converter at frequency ranges of 7.42–7.6 GHz and 13–13.56 GHz.

## Structure design

**Figure 1:**
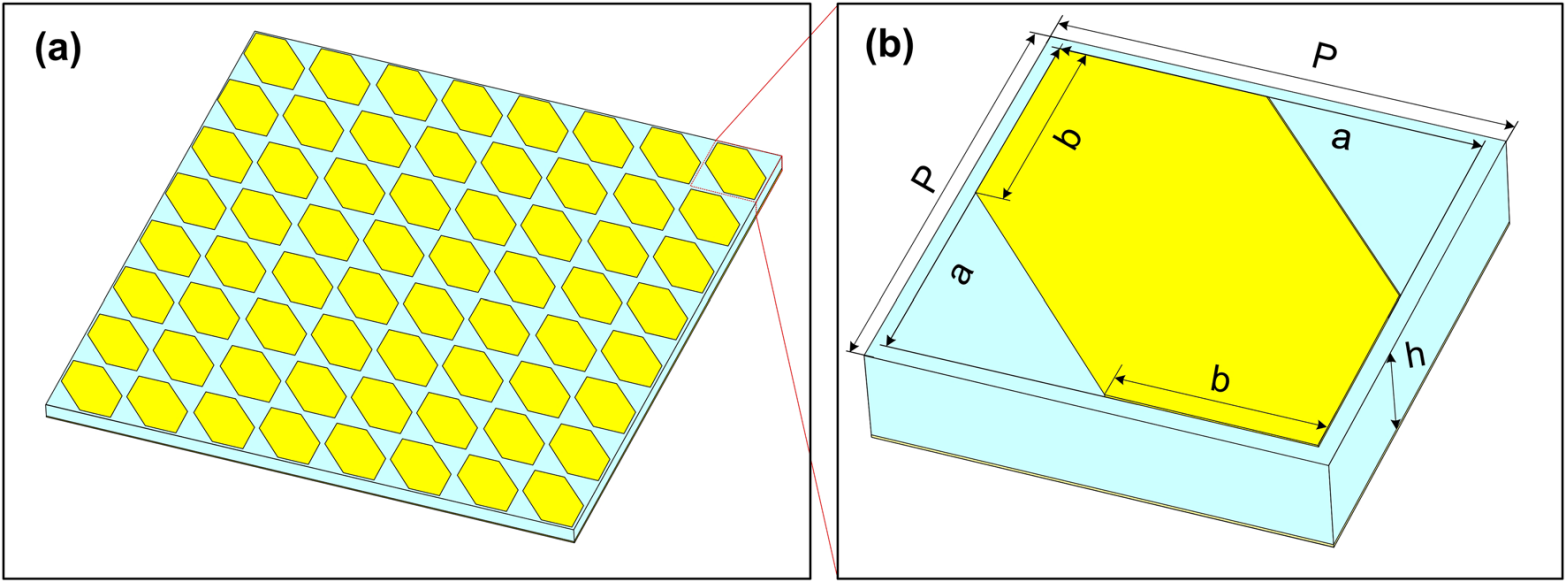
Schematic of the proposed polarization: (**a**) tilted top-view and (**b**) 3D-view of a unit cell.

Figure 1 shows a schematic of the proposed polarization converter (Fig. 1a) with a magnified unit cell (Fig. 1b). The converter structure consists of a periodic array of an anisotropic metasurface. The unit cell of the proposed converter is composed of a metallic two-corner-cut square patch resonator and a metallic ground plane separated by an FR-4 dielectric substrate with a thickness (*h*) of 1.6 mm. The top and the bottom layers are made by copper with an electric conductivity of $5.96\times 10^7$ *S/m* and a thickness of 0.035 mm. The dielectric substrate has a relative dielectric constant of 4.3 and a loss tangent of 0.025. The geometrical parameters of the unit cell are given by *P* = 6.92 mm, *b* = 3.17 mm, *a* = 6.4 mm, as shown in Fig. 1b.

To evaluate polarization conversion performance of a linear polarization converter, the polarization conversion ratio (PCR) can be used. Assuming the polarization of the incident electric field is along to the *y*-axis, the PCR is calculated as Eq. (1)^18,43^. Similarly, for *x*-polarized incident wave, the subscripts *x* and *y* are interchanged in Eq. (1).

1 $$\begin{aligned} PCR = \frac{\left| r_{xy}\right| ^{2}}{\left| r_{xy}\right| ^{2} +\left| r_{yy}\right| ^{2}} \end{aligned}$$

where $r_{xy}=\left| E_{rx}\right|$/$\left| E_{iy}\right|$ and $r_{yy}=\left| E_{ry}\right|$/$\left| E_{iy}\right|$ are reflection coefficients for cross-polarization and co-polarization, respectively. *E* indicates the electric field while *i* and *r* correspondingly refer to the incident and reflected EM wave. Due to the symmetric of the unit cell along the diagonal direction, the co-polarized reflection wave and cross-polarized reflection wave do not change about the magnitude and phase when the incident wave is along to the *x*-axis or *y*-axis^18,36^.

Similarly, the polarization maintaining ability of a circular polarization converter is determined by polarization maintaining ratio (PMR). The PMR for right-handed circularly polarized (RHCP) wave is shown in Eq. (2)^40,43^.

2 $$\begin{aligned} PMR = \frac{\left| r_{++}\right| ^{2}}{\left| r_{-+}\right| ^{2} +\left| r_{++}\right| ^{2}} \end{aligned}$$

where, the subscripts + and − denote RHCP and left–handed circular polarization (LHCP) states, respectively. For LHCP wave, the subscripts + and − are interchanged in Eq. (2).

Meanwhile, the normalized ellipticity (*e*) is used to evaluate the degree of circularly reflected wave, which is calculated by using Eq. (3) for the incident *y*-polarized wave^43^.

3 $$\begin{aligned} e = \frac{2\left| r_{xy}\right| \left| r_{yy}\right| \sin \Delta \Phi }{\left| r_{xy}\right| ^{2}+\left| r_{yy}\right| ^{2}} \end{aligned}$$

where, $\Delta \Phi =\Phi _{yy}-\Phi _{xy}$ with $\Phi _{yy}$ and $\Phi _{xy}$ are reflection phases for co-polarization and cross- polarization, respectively. When the normalized ellipticity (*e*) is + 1, which corresponds to $r_{xy}=r_{yy}$ and $\Delta \Phi =90^{\circ }+2k\pi$ (*k* is an integer), the reflected wave is LHCP. By contrast, in the case of the normalized ellipticity (*e*) is $-1$, which corresponds to $r_{xy}=r_{yy}$ and $\Delta \Phi =-90^{\circ }+2k\pi$ (*k* is an integer), the reflective wave is RHCP. Furthermore, axial ratio (AR) is proposed to measure the circular polarization which can be expressed as Eq. (4) for the incident *y*- and *x*-polarized wave^31,37,40^.

4 $$\begin{aligned} AR = 10log\frac{\left| r_{xy}\right| }{\left| r_{yy}\right| } =10log\sqrt{\frac{1-cos\Delta \Phi }{1+cos\Delta \Phi }} \end{aligned}$$

When the phase difference is $\Delta \Phi = \pm 90^{\circ }+2k\pi$ and $\left| r_{xy}\right| = \left| r_{yy}\right|$, the axial ratio is $\textit{AR}= 0$ dB, the reflected wave would be converted into a circular polarized wave.

## Results and discussion

**Figure 2:**
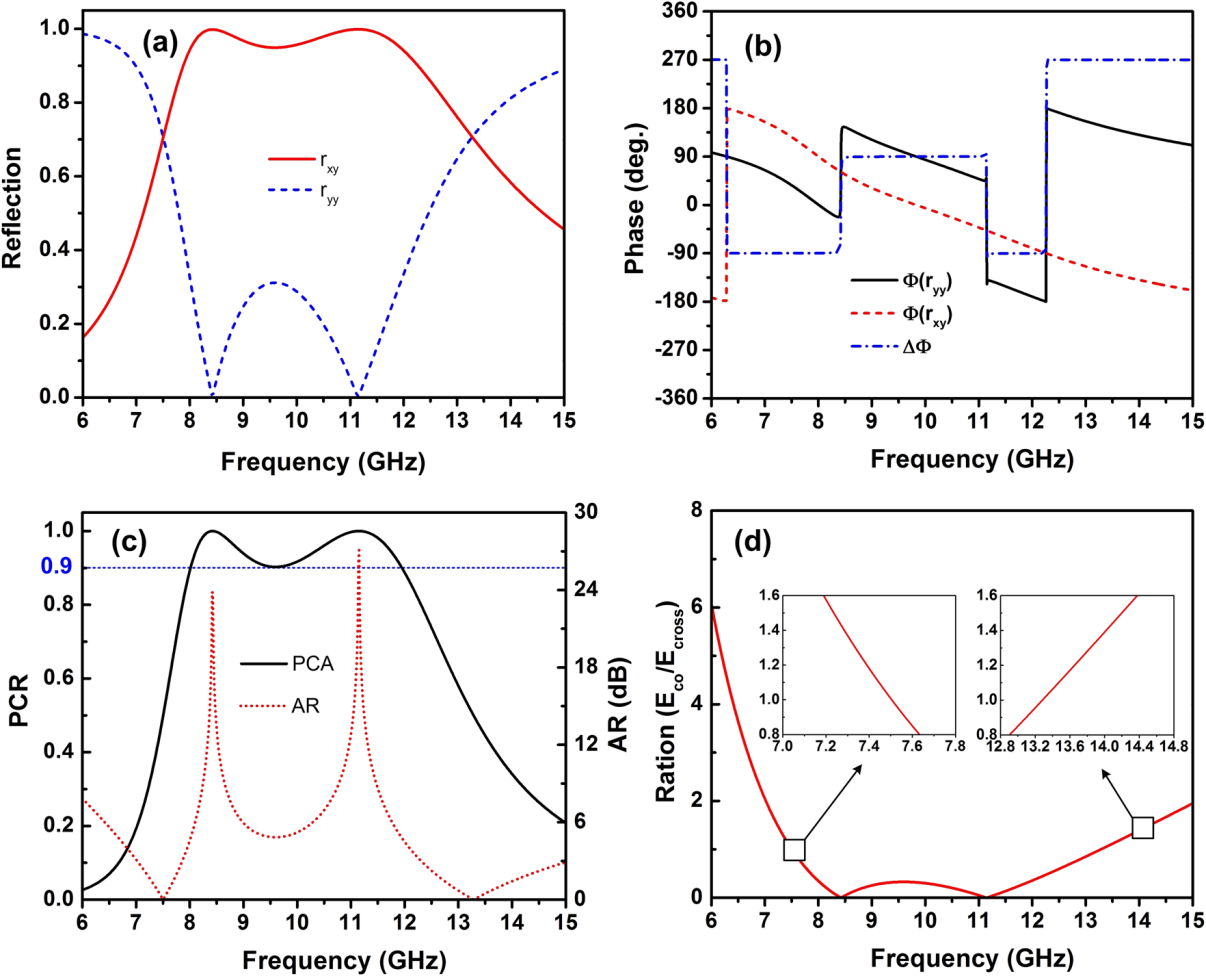
(**a**) Magnitude and (**b**) phase of co- and cross-polarized reflection coefficients, (**c**) PCR and AR, and (**d**) ratio of the co- and cross-polarized reflected fields of the proposed polarization converter under normal incidence for y-polarization.

Figure 2 presents the simulated results for the incident y-polarized wave under normal incidence. As shown in Fig. 2a, the magnitude of the cross-polarization coefficient ($r_{xy}$) is higher than 0.96, while the co-polarization coefficient ($r_{yy}$) is below 0.3 in the frequency range from 8 GHz to 12 GHz. The phase of reflection waves and their phase difference are shown in Fig. 2b. Although the phase difference ($\Delta \Phi$) changes from $-180^{\circ }$ to $270^{\circ }$, it does not affect the efficiency of the polarization converter following Eq. (1). That is explained by the magnitude value of $r_{xy}$ is significantly higher than $r_{yy}$ in the whole band. Therefore, the *PCR* is greater 0.9 in the frequency range of 8–12 GHz, as shown in Fig. 2c, which covers the entire X-band of 8–12 GHz. It indicates that the proposed converter operates efficiently as a linear polarization converter in the entire X-band. We note that the *PCR* reaches roughly the value of 1 at resonance frequencies 8.4 GHz and 11.2 GHz. Importantly, the incident y-polarized wave is reflected with the unity magnitude $r_{xy} = r_{yy}$ and phase differences are $-90^{\circ }$ and $270^{\circ }$ at two frequencies 7.5 GHz and 13.2 GHz, respectively. The *AR* is equal to 0 at these frequencies of 7.5 GHz and 13.27 GHz and lower than 3 dB in frequency range from 7 to 7.85 GHz and 12.3 to 14.97 GHz as depicted in Fig. 2c. This means the reflected wave is the right-handed circularly polarized at two frequencies of 7.5 GHz and 13.27 GHz. The frequency range with *AR* below 3 dB was considered to determine the operation bandwidth of a circular polarization converter^32^. Furthermore, the performance of a circular polarization converter can be estimated accurately if the ratio between the magnitude of the co-polarized and cross-polarized reflected fields is within the range of 0.85-1.15 and their phase difference is within the range from $n90^{\circ }-5^{\circ }$ to $n90^{\circ }+5^{\circ }$ where *n* is an odd integer^42–44^. Therefore, as shown in the inset of Fig. 2(d), the proposed converter also exhibits the circular polarization conversion in frequency ranges of 7.42-7.6 GHz and 13-13.56 GHz. At these frequency ranges, the *AR* is below 1 dB.

**Figure 3:**
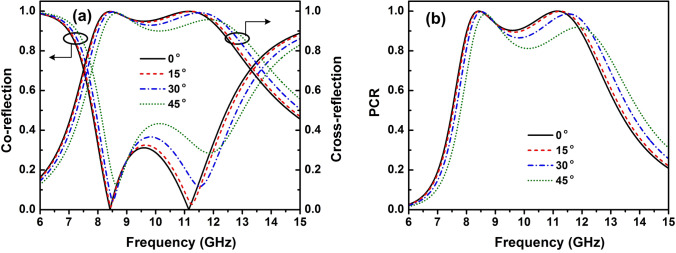
(**a**) Magnitude of co- and cross-polarized reflection coefficients and (**b**) PCR of the proposed polarization converter under incident angles ranging from $0^{\circ }$ to $45^{\circ }$ for TE polarization.

In practical applications, the EM wave is obliquely incident onto the surface of the designed structure; therefore, the incident angle stability of polarization converter should be considered. Figure 3a shows the dependence of the magnitude of the co- and cross-polarized reflection coefficients on the incident angle under transverse electric (TE) polarization. The magnitude of the co-reflection coefficient reduces while the magnitude of the cross-reflection coefficient increases with increasing of the incident angle. However, the PCR can be maintained above 0.8 in the operating frequency range from 8 to 12 GHz with incident angle up to $45^{\circ }$ (Fig. 3b).

**Figure 4:**
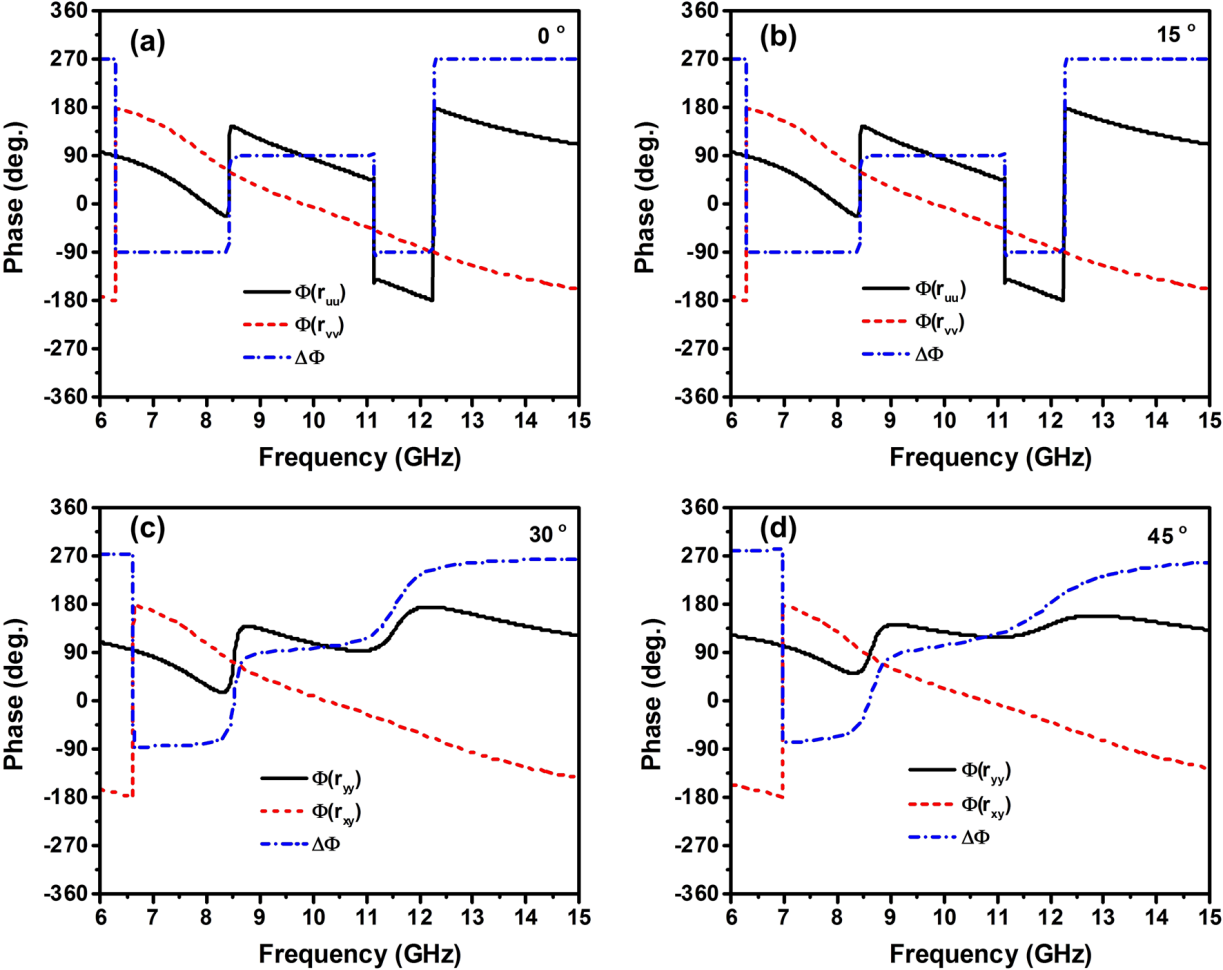
Phase of co- and cross-polarized reflection coefficients and their phase difference of the proposed polarization converter for various incident angles.

Figure 4 depicts the phase of the co- and cross-polarized reflection coefficients and their difference at different oblique incident angles for TE polarization. As seen in Fig. 4, the proposed design can achieve CPC with the incident angle of up to $45^{\circ }$ within a certain frequency bandwidth while the LP-to-CP conversion maintains angularly stable with the incident angle of up to $30^{\circ }$. It should be noted that the proposed polarization converter has the thickness of the dielectric substrate of $0.053\lambda$ and the unit cell size of 0.23$\lambda$ at the center of resonant frequency ($\lambda$) of the operating bandwidth of 10 GHz. These dimensions are much smaller than the operating wavelength, which can be attributed to the wide incident angle stability of the proposed converter. It was recently reported that the design of metasurface with its dimension smaller than the operating wavelength could improve angular stability^43^.

**Figure 5:**
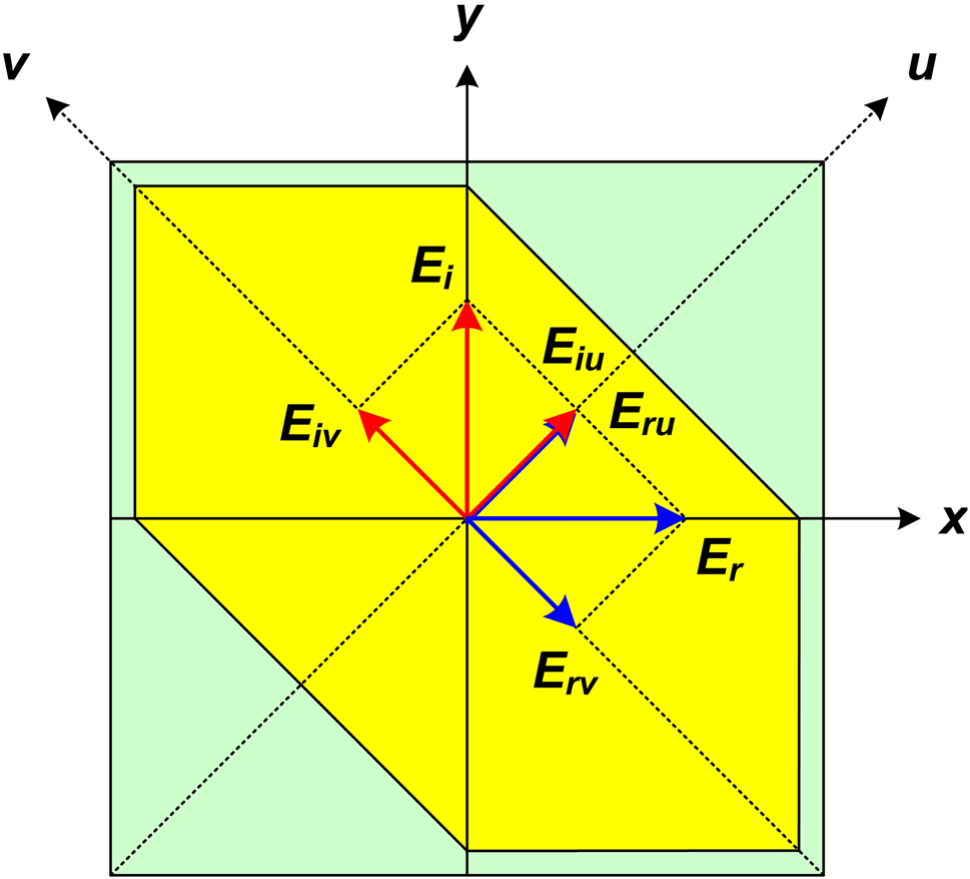
Working principle of the polarization converter.

The working principle of the proposed polarization converter can be explained as in Fig. 5. Assuming that incident EM wave (${{\varvec{E}}}_{i}$) is along to the *y*-axis, decomposed into two orthogonal *u*- and *v*-components. The *u*- and *v*-axes are inclined $45^{\circ }$ and $-45^{\circ }$ to *y*-axis, respectively. The incident and reflected wave can be given as Eqs. (5) and (6), respectively.

5 $$\begin{aligned} {{\varvec{E}}}_{i}= & {} {\widehat{y}}E_{i}={\widehat{u}}E_{iu} +{\widehat{v}}E_{iv} \end{aligned}$$

6 $$\begin{aligned} {{\varvec{E}}}_{r}= & {} {\widehat{u}}E_{ru}+{\widehat{v}}E_{rv} ={\widehat{u}}r_{u}E_{iu}+{\widehat{v}}r_{v}E_{iv} \end{aligned}$$

where ${\widehat{u}}$ and ${\widehat{v}}$ are the unit vectors and $r_{u}$ and $r_{v}$ are the complex reflection coefficients in the *u*- and *v*-axis, respectively.

Equation (7) can be written as proposed by Khan et al. as follows^43^:

7 $$\begin{aligned} {{\varvec{E}}}_{r}={\widehat{u}}(r_{uu}E_{iu}e^{i\Phi _{uu}} +r_{uv}E_{iv}e^{i\Phi _{uv}})+{\widehat{v}}(r_{vv}E_{iv}e^{i\Phi _{vv}} +r_{vu}E_{iu}e^{i\Phi _{vu}}) \end{aligned}$$

where, $r_{uu}$ and $r_{uv}$ and $r_{vv}$ and $r_{vu}$ are magnitude of co- and cross-reflection coefficients, $\Phi _{uu}$ and $\Phi _{vu}$ and $\Phi _{vv}$ and $\Phi _{uv}$ are phase of co- and cross-reflection coefficients in the *u*- and *v*-axis, respectively.

Due to the asymmetric structure, the proposed converter obeys anisotropic characteristics with dispersive relative permittivity and permeability^18,32,36^. Therefore, the difference in phase and magnitudes of reflection wave can be occurred. If $r_{uu}=r_{vv}\approx 1$, $r_{uv}=r_{vu}\approx 0$, and $\Delta \varphi =\Phi _{uu}-\Phi _{vv}=\pm 180^{\circ }+2k\pi$ (*k* is integer), the synthetic fields of $E_{ru}$ and $E_{rv}$ will be along the *x*-axis as shown in Fig. 5. It means that the incident polarized wave is rotated $90^{\circ }$ and the CPC converter occurs. In other case, if $\Delta \varphi =\Phi _{uu}-\Phi _{vv}=\pm 90^{\circ }+2k\pi$ (*k* is integer), the LP-to-CP converter can be obtained^43^.

**Figure 6:**
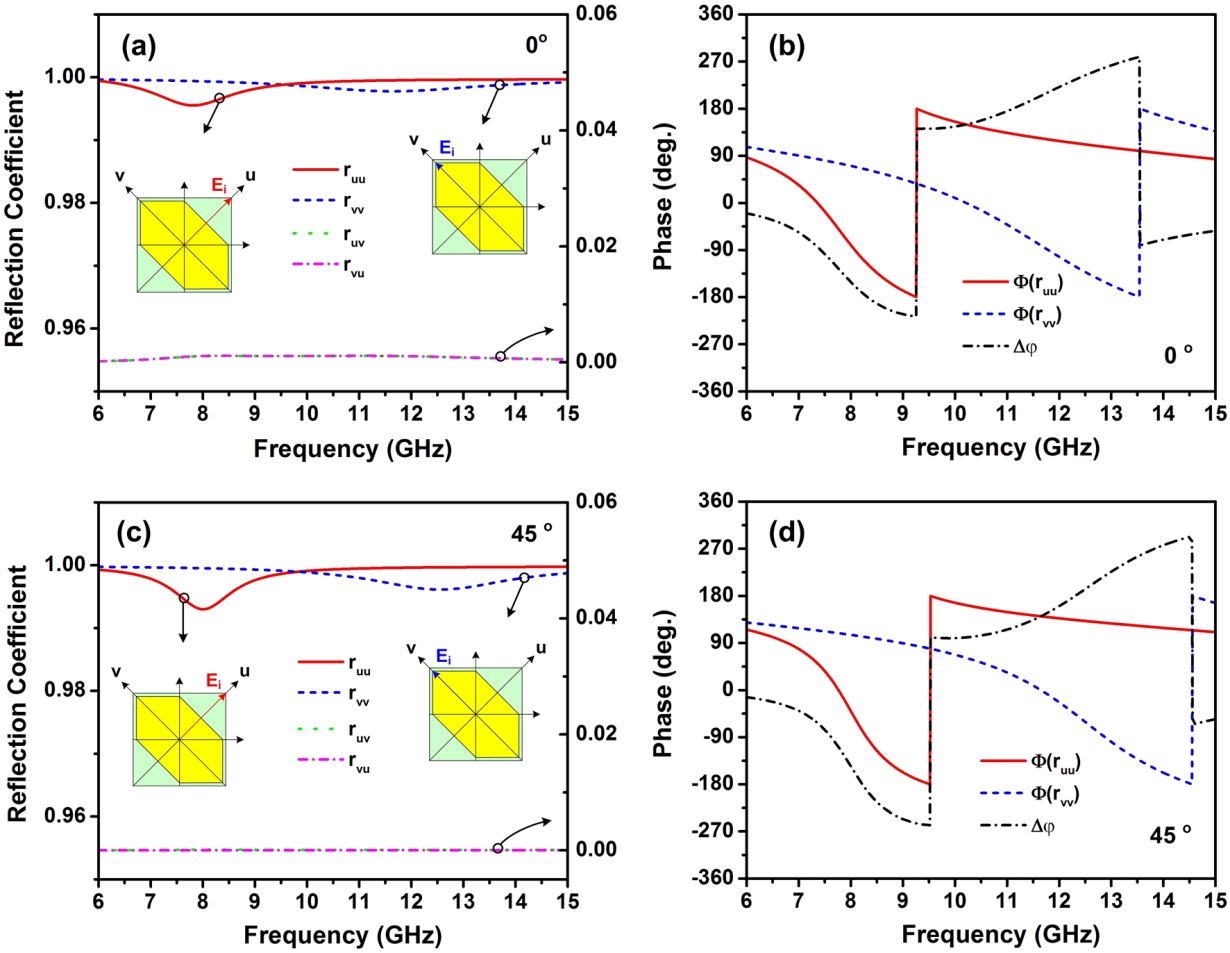
(**a**, **c**) Magnitude and (**b**, **d**) phase of the reflection coefficients of *u*- and *v*-components for the proposed polarization converter at the normal and $45^{\circ }$ incidence, respectively.

To evaluate the proposed converter property, the magnitude and phase response of the reflection coefficients of* u*- and *v*-components as a function of frequency are simulated. Figure 6a, **c** show the magnitude of reflection coefficients at normal and $45^{\circ }$ incidence, respectively. In both cases, the magnitude of the cross-polarized reflection coefficients is almost zero, while the co-polarized reflection coefficients are roughly equal to one for almost frequencies. Moreover, it can be seen in Fig. 6b, d, the phase differences between the reflected *u*- and *v*-polarized wave in the entire frequency range from 8 to 12 GHz is roughly $180^{\circ }$ or $-180^{\circ }$ indicates that the designed converter exhibits the linear cross-polarization conversion in a broad spectrum. Meanwhile, at frequency bands of 7.42–7.6 GHz and 13–13.56 GHz, the phase difference is approximate $-90^{\circ }$ or $270^{\circ }$, which means that the proposed design reveals the LP-to-CP conversion.

**Figure 7:**
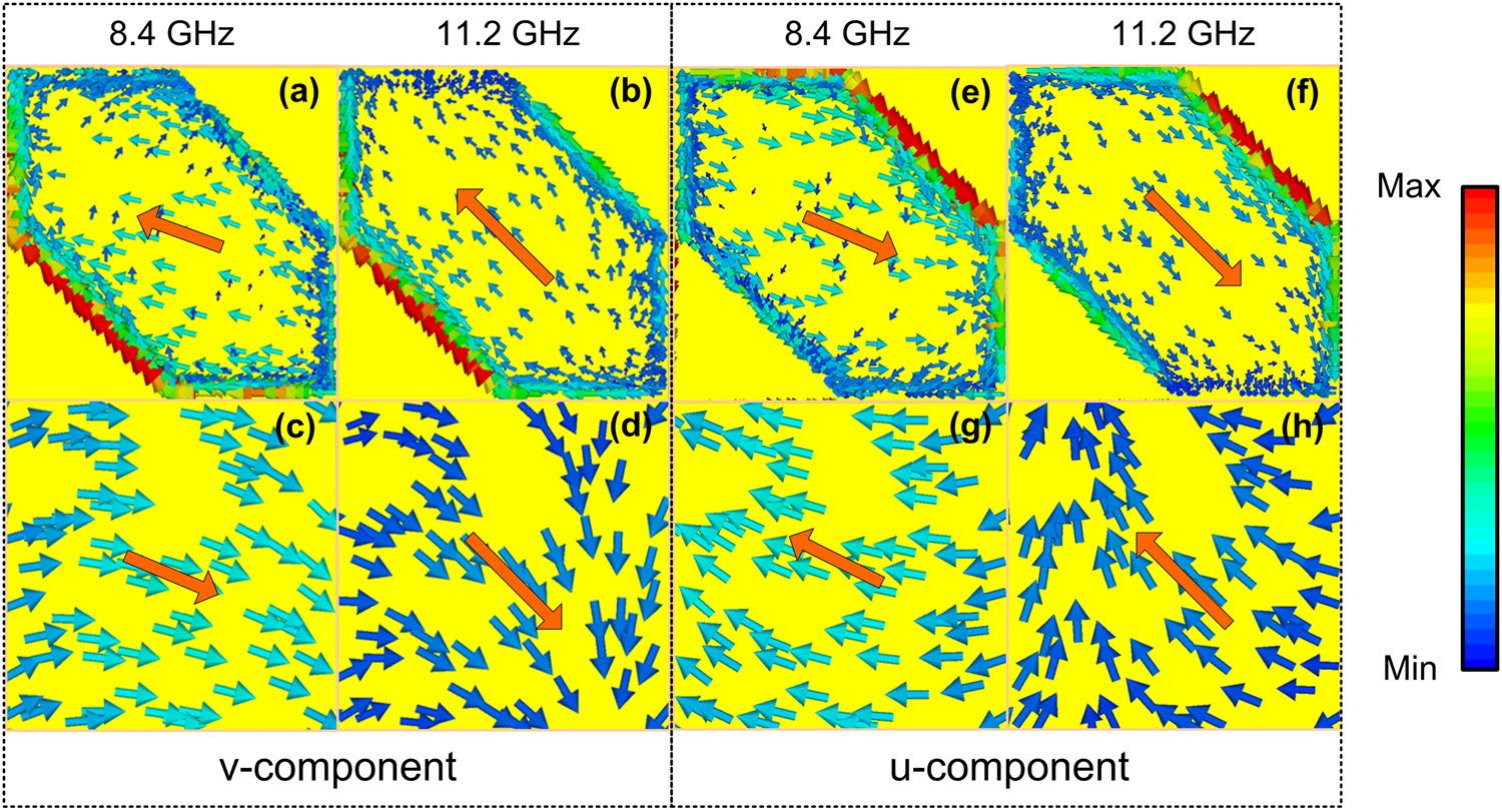
Distributions of surface current on (**a**), (**b**), (**e**), (**f**) the top layer and (**c**), (**d**), (**g**), (**h**) bottom layer of a unit cell for normal incidence in response to the *v*- and *u*-components with various resonant frequencies of 8.4 GHz and 11.2 GHz, respectively.

To further understand the physical mechanism, surface current distributions on the top and bottom metallic layer of the proposed converter in response for *u*- and *v*-components are simulated at the resonant frequencies of 8.4 GHz and 11.2 GHz as shown in Fig. 7. It is clear from Fig. 7, the surface currents at the top layer are anti-parallel to the bottom surface current for both *u*- and *v*-components. Thus, the current loops are created in the dielectric layer and formed the magnetic resonance. It was reported that the combination of PCRs around resonance frequencies is main reason for achieving high efficiency and broadband polarization converter^18^.

**Figure 8:**
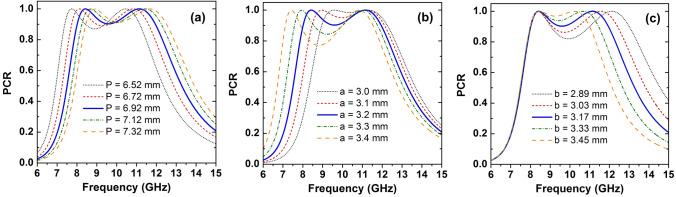
The variation of PCR with various unit cell parameters of the proposed polarization converter: (**a**) *P*, (**b**) *a*, and (**c**) *b*.

We have carried out a parametric study to analyze the effect of geometrical parameters on the performance of the proposed polarization converter. Based on this analysis, we can reach the optimized structure shown in Fig. 1. The PCR of the proposed converter is simulated at different periods (*P*) in the range of 6.52–7.32 mm while other parameters are kept constant, as shown in Fig. 8a. When *P* increases, the operating frequency band is shifted to higher frequency while the polarization conversion is slightly increased. For obtaining operating bandwidth located in the entire X-band of 8-12 GHz, the *P* is chosen at 6.92 mm. Once the *P* is determined, the PCR for various parameters of *a* in the range of 3.0–3.4 mm and *b* in the range of 2.89–3.45 mm are simulated, as illustrated in Fig. 8b, c. With increasing *a* and *b*, the operating bandwidth is extended, however, the polarization conversion is decreased. The designed parameters are determined while considering both operating bandwidth and performance. Therefore, the *a* and * b* are optimized at 3.2 mm and 3.17 mm for achieving the highest polarization conversion and widest bandwidth, respectively.

**Figure 9:**
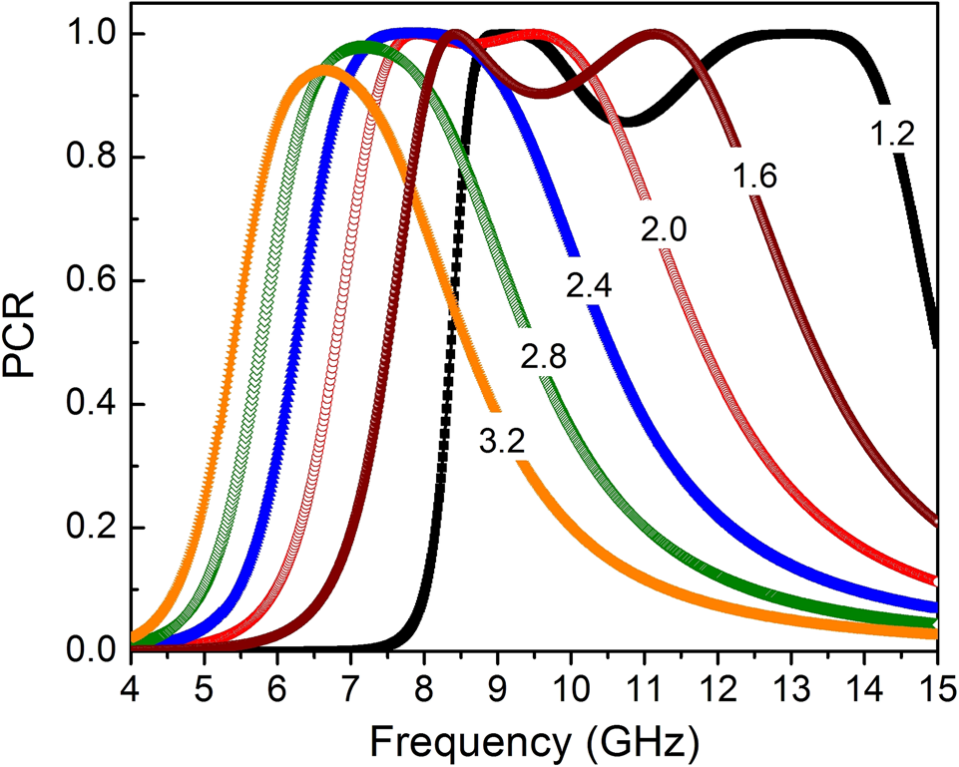
The PCR of the proposed polarization converter as a function of thickness of dielectric layer.

**Figure 10:**
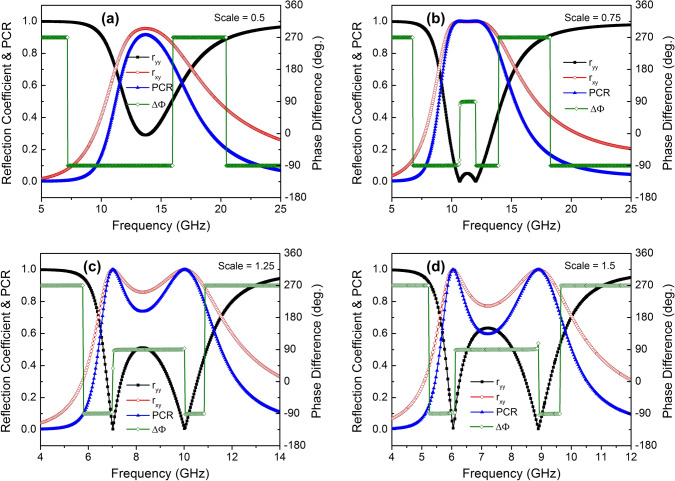
Magnitude of co- and cross-polarized reflection coefficients, phase difference, and PCR of the proposed polarization converter for different scaled physical dimensions in xy-plane: (**a**) 0.5, (**b**) 0.75, (**c**) 1.25, and (**d**) 1.5.

Furthermore, to tailor the operating frequency band satisfied the requirements of a specific application, we have also simulated the variation of the thickness of dielectric layer and unit cell dimension of the proposed converter. Figure 9 shows PCR of the proposed converter as a function of the thickness in the range of 1.2 mm to 3.2 mm while other parameters kept constant. It is clear that the operating frequency of the proposed converter shifts to the lower frequency range by increasing the thickness of dielectric layer. Furthermore, the variation of operating frequency band of the proposed converter is also investigated with the unit cell scaling from 0.5 to 1.5 with step of 0.25 in the *xy*-plane. As shown in Fig. 10a, in case of scaling of 0.5, the operating frequency band of the converter for CPC shifts from 8–12 to 12.89–14.5 GHz, whereas the operating frequency for LP-to-CP increases significantly from 7.5 and 13.2 GHz to 11.13 and 17.5 GHz, respectively. Similarly, the operating frequency of LP-to-CP changes to 8.95 GHz and 15.32 GHz, and the frequency band for CPC shifts to 9.73–13.47 GHz when the dimension of the unit cell is scaled by 0.75 (Fig. 10b). Furthermore, with increasing the physical dimension by scaling of 1.25 and 1.5, the operating frequencies of the polarization for CRC changes to the lower frequency regime, in ranges 6.78–10.58 GHz and 5.87–9.32 GHz, as seen in Fig. 10c, d, respectively. Likewise, the operating frequencies for LP-to-CP transformation decrease to 6.4 GHz and 11.52 GHz and 5.61 GHz and 10.04 GHz, respectively. Based on the above examination, we emphasize that the operating frequency of the proposed converter can be control to work in suitable frequency regimes for practical applications through adjusting parameters of the unit cell metasurface or the substrate thickness.

Finally, the comparison of the performance between our proposed polarization converter and other broadband polarization converter is studied. For comprehensive comparison, the cost-efficient bandwidth ($BW_{CE}$) is introduced as Eq. (8)^45^.

8 $$\begin{aligned} BW_{CE}=\frac{RBW}{R_{V}} \end{aligned}$$

where *RBW* is relative bandwidth and $R_{V}=T_{V}/\lambda ^{3}$ is the relative volume of a unit cell. $T_{V}$ is the total volume of a unit cell and $\lambda$ is the wavelength of the center frequency of the operation bandwidth of polarization converter. The polarization converter characteristics in terms of the resonant frequency range, polarization conversion property, *RBW*, thickness, and $BW_{CE}$ are shown in Table 1. It can be observed that the proposed converter has a competitive design characterized by simple structure, compact size, multifunctional property, and the large cost-efficient bandwidth and wide incident stability in the cross-polarization conversion.

**Table 1:** Comparison the performance of the proposed polarization converter with other broadband polarization converter.

Ref	Operating bandwidth (GHz)	Polarization converter properties	Angular stability (deg.)	RBW with PCR $>90\%$	Thickness (mm)	Length of a unit cell (mm)	$\hbox {BW}_{{CE}}$
12	6.67–17.1	CPC	NA	87.7	3.5 (0.139$\lambda$)	12 (0.48$\lambda$)	27.38
13	8.9–11.1	CPC	30	22	1.27 (0.042$\lambda$)	5.7 (0.19$\lambda$)	145.1
25	9.4–19.2	CPC	NA	68.5	3.0 (0.095$\lambda$)	10 (0.48$\lambda$)	31.3
26	5.7–10.3	CPC	45	57.5	2.9 (0.077$\lambda$)	10 (0.27$\lambda$)	102.44
37	8.0-11.0	CPC	45	31.6	1.6 (0.05$\lambda$)	7.0 (0.22$\lambda$)	130.58
	5.7–7.7 & 11.5–11.9	LP-to-CP	45	2.6 & 3.4			10.74 & 14.05
This work	8.0–12.0	CPC	45	40	1.6 (0.053$\lambda$)	6.92 (0.23$\lambda$)	142.67
	7.42–7.6 & 13–13.56	LP-to-CP	30	2.4 & 4.2			8.56 & 14.98

**Figure 11:**
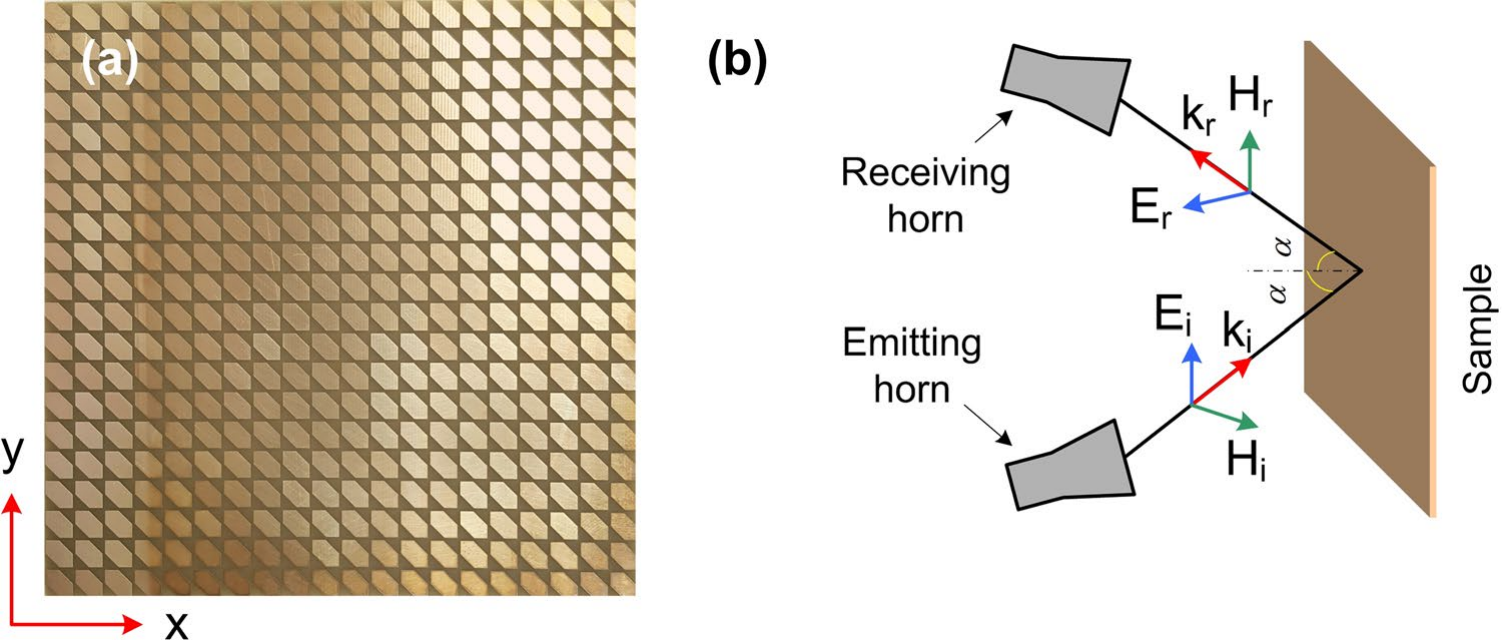
(**a**) Photograph of the fabricated sample and (**b**) schematic illustration of the measurement setup.

## Experimental verification

**Figure 12:**
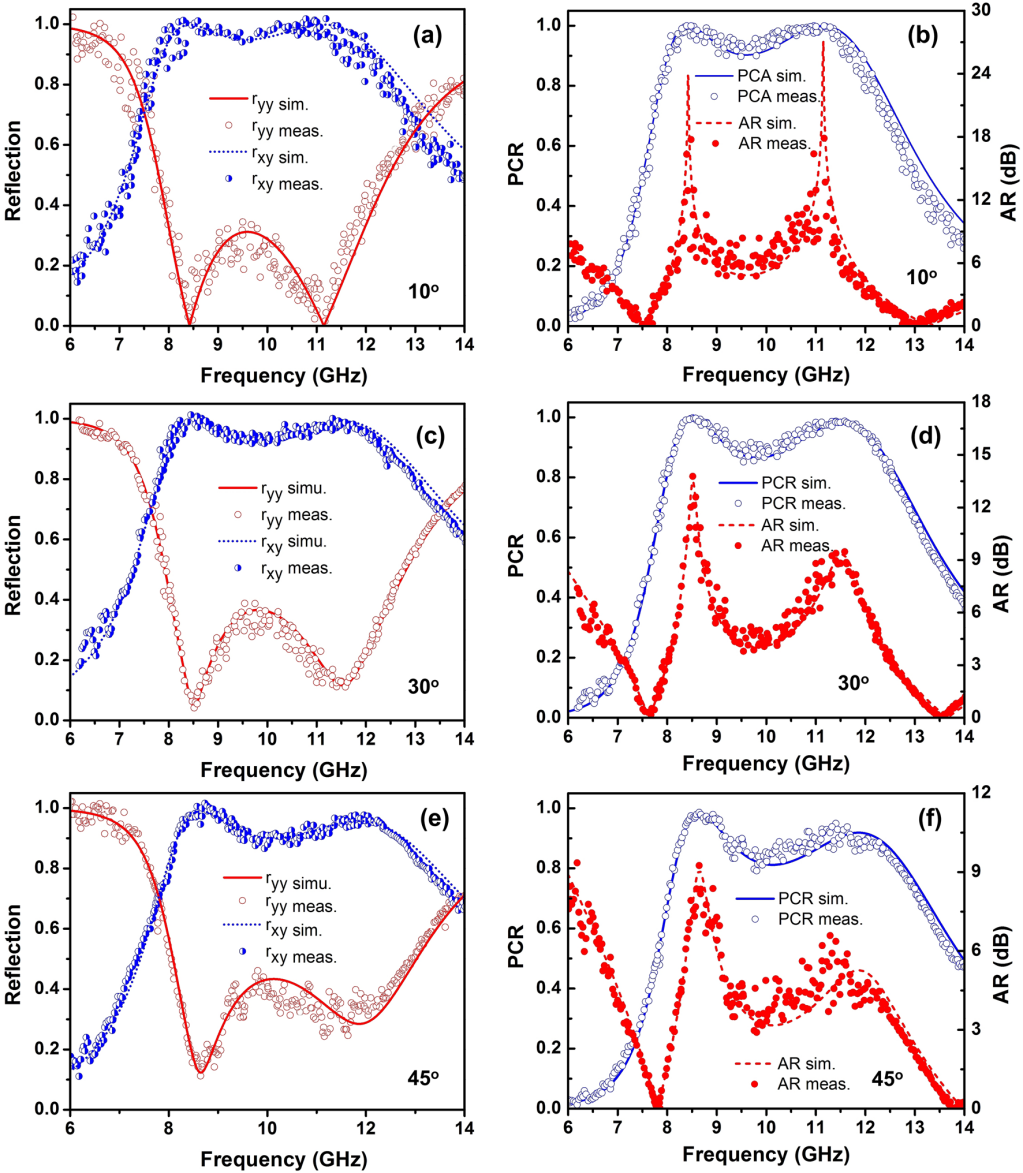
(**a**, **c**, **e**) Magnitude of simulated and measured co- and cross-polarized reflection coefficients and (**b**, **d**, **f**) *PCR* and *AR* of the proposed polarization converter under different incident angles of 10$^{\circ }$, 30$^{\circ }$, and 45$^{\circ }$, respectively.

To verify the performance of the designed polarization converter, device fabrication was using the conventional printed circuit board process, which has the structural parameters the same as the simulated model. The fabricated sample image is shown in Fig. 11a, which has an oversize of 138.4 mm $\times$ 138.4 mm and contains 20 $\times$ 20 unit cells. The unit cell parameters of fabricated sample are *P* = 6.92 mm, *a* = 3.2 mm, *b* = 3.17 mm, and *h* = 1.6 mm. To analyze the performance of proposed converter, the reflection coefficients as function of frequency were measured by the Rohde and Schwarz ZNB20 vector network analyzer together with two identical linearly polarized standard-gain horn antennas as transmitter and receiver, as shown in Fig. 11b. One horn antenna is used to emit* x*- or *y*-polarized waves, and the other horn antenna is used to receive *x*- and *y*-polarized waves. The co- and cross-polarized reflection coefficients are measured through the vector network analyzer. Figure 12 shows the measured magnitude of reflection spectra and *PCR* and *AR* with various oblique angle of $10^{\circ }$, $30^{\circ }$, and $45^{\circ }$, respectively. It can observed that the experimental results are in good agreement with the simulation results. Slight differences between the simulation and measurement are attributed to experimental factors such as the imperfections in the fabrication process, system alignment errors, and the limited size of the fabricated sample^3,18,43^. At nearly normal incidence (oblique angle below $10^{\circ }$), the measured magnitude of co-polarized reflection coefficient is higher than 0.9 and the measured magnitude of cross-polarized reflection coefficient is lower than 0.3 in the range of 8–12 GHz, thus the corresponding measured *PCR* is higher than 0.9. It is indicated that the proposed polarization converter is a highly efficient linear polarization conversion in the range of 8–12 GHz. Also, the measured *AR* is 0 at the frequencies of 7.5 GHz and 13.27 GHz and below 1 dB at frequency ranges of 7.42–7.6 GHz and 13–13.56 GHz, which means the circular polarization conversion. Moreover, the measured *PCR* is decreased, however, it can keep higher than 80% in the range of 8–12 GHz with increasing incident angle up to $45^{\circ }$, which confirms the wide incident angle insensitivity.

## Methods

The commercial CST-Microwave Studio software with a frequency-domain solver is used to investigate the performance of the designed converter. In this study, the periodic boundary conditions are fixed to unit cell for *x* and *y* directions and open for *z*-direction. The incident wave (*k*) is polaried along-*z* direction. The simulation is performed in free space.

The material used in the device fabrication is the copper coated FR-4 substrate on both sides with a copper thickness of 0.035 mm and an electric conductivity of $5.96\times 10^7$ *S/m*. The thickness of FR-4 substrate is 1.6 mm and the relative dielectric constant is 4.3. The fabricated sample with an oversize of 138.4 mm $\times$ 138.4 mm is fabricated by photolithography technique using a halogen light source with power of 45 W. The resolution of fabrication system is 0.01 mm.

The the reflection coefficients of the design converter are measured by the Rohde and Schwarz ZNB20 vector network analyzer with two identical linearly polarized standard-gain horn antennas as transmitter and receiver. The separation angle between two antennas is set to be $10^{\circ }$, corresponding to the normal incidence measurement in the experimental. The co- and cross-polarized reflection coefficients are measured through the rotation of receive antenna by $0^{\circ }$ and $90^{\circ }$, respectively.

## Conclusion

We have successfully realized a linear and linear-to-circular polarization converter simultaneously based on the metasurface structure with the two-corner-cut square patch resonance, working in the X-band frequency range. The proposed converter achieves a high CPC efficiency above 90% in the wide bandwidth range from 8 to 12 GHz. This efficiency can be maintained higher than 80% with a large incident angle up to 45°. Furthermore, the LP-to-CP conversion is retained in the frequencies ranges of 7.42–7.6 GHz and 13–13.56 GHz with the incident angle stability up to 30°. The prototype of proposed converter was fabricated and measured. The experimental result shows a good agreement with the simulated results. Compared with previously reported broadband converters, the proposed design presents high practical feasibility in terms of simple structure and high performance with the large cost-efficient bandwidth and wide incident stability in the linear cross-polarization conversion, suggesting its promising application for X-band applications. Besides, we believe that this structure can be enlarged to design broadband polarization converters in other frequency bands.

## Data Availability

The datasets generated during and/or analyzed during the current study are available from the corresponding author on reasonable request.
